# Supplementary material for: Eight-Year Health Risks Trend Analysis of a Comprehensive Workplace Health Promotion Program
Source: Int J Environ Res Public Health. 2020 Dec 16;17(24):9426. doi: 10.3390/ijerph17249426 (PMC7765570; doi:10.3390/ijerph17249426)
Supplement: Supplementary file 1 [file ijerph-17-09426-s001.zip › Supplementary S2. Implementation components.docx]

Supplementary S2. Implementation components of the three time-periods 2010–2013, 2014–2017 and 2010–2017

|  | PHASE 1  2010-2013 | PHASE 2  2014-2017 | TOTAL PERIOD  2010-2017 |
| --- | --- | --- | --- |
| The main focus of the program | pleasant lifestyle change, nutrition, physical activity | workplace climate, stress management, mental resources | both mentioned |
| Population (n) | 359 | 255 | 253 |
| The same participants | 215 | 215 | 215 |
|  |  |  |  |
| Planned actions | 73 | 88 | 161 |
| Implemented actions | 59 | 69 | 128 |
|  |  |  |  |
| **Program components** |  |  |  |
| Health risk assessment | x | x | x |
| Targeted services | 27 | 49 | 76 |
| Services for all employees | x | x | x |
| Playmaker network | x | x | x |
| Communication tools | x | x | x |
| Process management | x | x | x |
|  |  |  |  |
| **PIPE Impact Metric*** |  |  |  |
| Penetration | 0.992 | 0.952 | 0.972 |
| Implementation | 0.808 | 0.784 | 0.795 |
| Participation | 0.490 | 0.687 | 0.779 |
| Effectiveness | 0.463 | 0.279 | 0.273 |
| Total Health Impact | 18.2% | 14.3% | 16.4% |

**PIPE Impact Metric model is based on a scientific framework developed by Pronk (2001) and the calculations are derived from the analysis of the exact same comprehensive program and cohorts (Äikäs et al 2019). Each of the PIPE Impact Metric coefficient has own calculation methodology and the values vary from 0.000 to 1.000 (Pronk 2001). Total Health Impact is calculated by multiplying each coefficient: Penetration x implementation x Participation x Effectiveness (Pronk 2001).*
