# Supplementary material for: Eight-Year Health Risks Trend Analysis of a Comprehensive Workplace Health Promotion Program
Source: Int J Environ Res Public Health. 2020 Dec 16;17(24):9426. doi: 10.3390/ijerph17249426 (PMC7765570; doi:10.3390/ijerph17249426)
Supplement: Supplementary file 1 [file ijerph-17-09426-s001.zip › Supplementary S3. Test for HRA participants.docx]

Supplementary S3. Similarity test for Health Risk Accumulation among Participant versus Non-participants

|  | BASELINE |  | COHORT1 |  |  | COHORT3 |  |
| --- | --- | --- | --- | --- | --- | --- | --- |
| Results at baseline | HRA Participants | HRA Participants | Non-participants | P-value* | HRA Participants | Non-Participants | P-value |
| N | 588 | 359 | 229 |  | 253 | 335 |  |
| Mean age (years) |  | 42.6±10.2 | 45.8±11.9 | 0.000^a^ | 41.2±9.6 | 45.9±11.5 | 0.000^a^ |
| Average number of health risks | 1.49±1.3 | 1.33±1.3 | 1.73±1.4 | 0.000^a^ | 1.25±1.3 | 1.67±1.4 | 0.000^a^ |
| Overall health risk level |  |  |  |  |  |  |  |
| Low | 58,8 % | 63,5 % | 51,5 % | 0.011^b^ | 67,6 % | 52,2 % | 0.001^b^ |
| Moderate | 32,5 % | 29,5 % | 37,1 % |  | 25,3 % | 37,9 % |  |
| High | 8,7 % | 7,0 % | 11,4 % |  | 7,2 % | 9,9 % |  |
|  |  |  |  |  |  |  |  |
|  |  |  |  |  |  |  |  |
|  |  |  |  |  |  |  |  |
|  | BASELINE |  | FOLLOW-UP1 |  |  | FOLLOW-UP2 |  |
| Results at baseline | HRA Participants | HRA Participants | Non-participants | P-value* | HRA Participants | Non-Participants | P-value |
| N | 588 | 366 | 222 |  | 317 | 271 |  |
| Mean age (years) |  | 42.7±10.2 | 45.8±11.9 | 0.001^a^ | 41.2±9.58 | 45.9±11.5 | 0.000^a^ |
| Average number of health risks | 1.49±1.3 | 1.34±1.3 | 1.73±1.4 | 0.001^a^ | 1.25±1.3 | 1.67±1.4 | 0.000^a^ |
| Overall health risk level |  |  |  |  |  |  |  |
| Low | 58,8 % | 63,1 % | 51,8 % | 0.019^b^ | 67,2 % | 49,1 % | 0.000^b^ |
| Moderate | 32,5 % | 29,8 % | 36,9 % |  | 25,6 % | 40,6 % |  |
| High | 8,7 % | 7,1 % | 11,3 % |  | 7,3 % | 4,8 % |  |
|  |  |  |  |  |  |  |  |

Results are based on the eight health risk measurements, which were similar for each measurement

^a^P-values are based on the independent samples T Test

^b^P-values are based on the Chi-square test
