# Supplementary material for: Eight-Year Health Risks Trend Analysis of a Comprehensive Workplace Health Promotion Program
Source: Int J Environ Res Public Health. 2020 Dec 16;17(24):9426. doi: 10.3390/ijerph17249426 (PMC7765570; doi:10.3390/ijerph17249426)
Supplement: Supplementary file 1 [file ijerph-17-09426-s001.zip › Supplementary S4. Participants completing HRAs.docx]

Supplementary S4. Participants who completed at least two HRAs and biometrical screenings 2010–2017, 2010–2013 and 2014–2017

Baseline → 1. Follow-up

1. Follow-up → 2. Follow-up

Baseline → 2. Follow-up

Baseline → 1. Follow-up → 2. Follow-up

2. Follow-up 2016-2017

1. Follow-up 2013-2014

Baseline 2010-2011

n=215

n=215

n=40

n=144

n=38
